# Management of elderly patients with acute promyelocytic leukemia: progress and problems

**DOI:** 10.1007/s00277-013-1788-z

**Published:** 2013-05-22

**Authors:** Eva Lengfelder, Wolf-Karsten Hofmann, Florian Nolte

**Affiliations:** 1 Department of Hematology and Oncology, University Hospital Mannheim, Mannheim, Germany; 2 III. Medizinische Klinik, Hämatologie und Onkologie, Universitätsmedizin Mannheim, Theodor-Kutzer-Ufer 1-3, 68167 Mannheim, Germany

**Keywords:** Acute promyelocytic leukemia, Elderly patients, Incidence, Early death, Treatment, Prognosis

## Abstract

Despite substantial progress in the management and outcome of acute promyelocytic leukemia (APL) during the last decades, older age remains a prominent negative prognostic factor. The improvement of long-term stabilization and cure of older APL patients is therefore a particular challenge. Data of unselected population-based studies suggest a high rate of exclusion from clinical trials in older age. The comparison of registry and study data indicates that study patients represent a positive selection. Older APL patients seem as sensitive to therapy as younger patients. With conventional therapy, based on all-*trans* retinoic acid (ATRA) and chemotherapy, over 50 % of older APL patients can probably be cured. Special problems of advanced age are the high rate of early death before or during induction therapy and the high frequency of death in remission with negative influence on the outcome. Both may be related in part to a higher vulnerability against the common treatment with ATRA and chemotherapy. Alternative less toxic approaches including arsenic trioxide (ATO) with or without ATRA and combinations with gemtuzumab ozogamicin or with reduced chemotherapy can induce long-lasting remission in all stages of APL. Considering the high curative potential and the excellent tolerance of ATO in newly diagnosed and relapsed APL, older patients are probably a particular target group for a chemotherapy-free approach with ATO.

## Introduction

Acute promyelocytic leukemia (APL) accounts for approximately 5–10 % of cases of acute myeloid leukemia (AML). It can be differentiated from all other forms of leukemia by its characteristic morphology (AML FAB M3/M3v) and by the diagnostic chromosomal translocation t(15;17) with corresponding PML-RARA and RARA-PML fusion genes [1–6]. Accompanying coagulation disorders promote the appearance of life-threatening hemorrhages, which are the most frequent cause of death in the early phase of the disease [7, 8]. Approximately 20 to 30 % of patients present with high white blood cell (WBC) counts associated with an inferior prognosis [9–11]. Other unfavorable prognostic factors are the expression of the CD56 antigen and the detection of FLT3 mutations [12–14]. Even in the presence of negative factors, the prognosis of APL still remains favorable in comparison to other AML subtypes if the patients receive the common treatment with all-*trans* retinoic acid (ATRA) and chemotherapy. The median age of patients included in larger trials is almost 40 to 45 years [15–23] (Table 1). With current treatment strategies, approximately 80 % of patients with APL included in trials can be cured [24, 25]. Older age, however, remains a striking negative prognostic factor despite improvement of prognosis over the last decades. The great majority of clinical reports on APL have focused on the progress of the younger age groups. It is the aim of the present review to give an overview on incidence, clinical characteristics, special problems, and treatment results of the elderly APL patients.

**Table 1:** Overview of the rate of older APL patients included in multicenter trials with ATRA and chemotherapy

Author (year) [reference]	Number of patients	Age (years), median (range)	≥60 years (%)	≥70 years (%)	Total patients with high risk (%)
Kanamaru (1995) [15]	109	43 (16–74)	–	7 (>69 years)	–
Burnett (1999) [16]	239	n.r. (<14–60+)	12	3	27
Tallman (2002) [18]	300	38 (1–81)	10 (>65 years)	–	–
Ades (2005) [19, 33]	533	46 (32–70)	24	6	–
Sanz (2010) [20]	963	41 (18–70+)	17	6	27
Burnett (2012) [21]	285	42 (16–69)	6	0	21
Lengfelder (2013) [22, 23]	260	51 (16–87)	26	9	28

*n*.*r*. not reported

## Incidence of APL in elderly patients and inclusion in clinical trials

It was primarily assumed that APL is less frequent in older patients. Based on data of the British Leukemia registries published in the year 2000, the incidence of APL was reported to be very low in children below the age of 10 years. Above this age limit, the incidence appeared approximately constant with increasing age [26]. Recently published results of other registries even show a significant increase of the incidence of APL in older age [27–30]. Details are shown in Table 2.

**Table 2:** Results of population-based studies indicating higher incidence of APL and higher early death rate with increasing age

Author (year) [reference]	Age groups (years)	No. of pts	Incidence per 100,000	Early death	*p* value	Outcome		*p* value
Park (2011) [28]				Probability of ED (%)		Survival rate (%)		
						1 year	3 years	
	<35	433	0.13	12.3	<0.001	81.2	76.3	<0.001
	35–54	463	0.26	16.0		75.6	72.0	
	≥54	502	0.42	24.2		53.2	46.4	
	Total	1,400						
Chen (2012) [29]				Probability of ED (%)		Cumulative relative survival		
						1 year	5 years	
	<20	149	0.06	n.r.		0.79	0.52	
	20–39	372	0.19	n.r.		0.71	0.57	
	40–59	427	0.22	n.r.		0.67	0.57	
	≥60	449	0.36	n.r.		0.38	0.24	
	Total	1,397						
Lehmann (2011) [27]				Rate of ED (%)		Survival rate (%)^a^		
						1 year	5 years	
	<40	28		19		82	82	
	40–59	37		16		83	74	
	≥60	40		50		37	24	
	Total	105						

*n*.*r*. not reported, *pts* patients, *ED* early death

^a^Approximate values extracted from survival curves

Compared to these unselected population-based studies, the rate of older patients included in clinical trials was mostly lower and ranged from 6 to 26 % in patients over 60 years and from 3 to 9 % at age over 70 years (Table 1) [15–23]. The percentage of elderly study patients assigned to the high-risk group, according to the Sanz score, varied considerably and was mostly lower compared to the total study populations covering all age groups (Tables 1 and 3) [23, 31–35]. These observations suggest that a considerable rate of elderly patients is not included in clinical trials, in particular those with high-risk disease (high risk according to Sanz score: WBC count >10,000/μl, intermediate risk: WBC count ≤10,000/μl and platelet count ≤40,000/μl, low risk: WBC count ≤10,000/μl and platelet count >40,000/μl) [9].

**Table 3:** Age distribution and treatment regimens in reports of elderly APL patients

Author (year) [reference]	Number of patients	Median age (range)	High risk (%)	Age ≥70 years (%)	Induction regimen	Consolidation cycles	Maintenance
Mandelli (2005) [31]	134	66 (60–75)	–	14	ATRA + Ida	1–3	Yes
Sanz (2004) [32]	104	68 (60–83)	20	33	ATRA + Ida	3	Yes
Ades (2005) [33]	129	66 (62–70)	–	0	ATRA + AD	1–2	Yes
Lataglia (2010) [34]	60	66 (60–73)	14	–	ATRA + Ida	1	Yes
Ono (2011) [35]	46	63 (60–70)	20	0	ATRA + Ida + Ara-C	3	Yes
Lengfelder (2013) [23]	56	67 (60–83)	32	32	ATRA + TAD (±HAM)	1	Yes

*ATRA* all-*trans* retinoic acid, *Ida* idarubicin, *ara*-*C* cytosine arabinoside, *AD* ara-C daunorubicin, *TAD* 6-thioguanine ara-C daunorubicin, *HAM* high-dose ara-C mitoxantrone

## Rate and reasons of early death in elderly APL patients

Despite improvement of prognosis in APL by the introduction of ATRA, the early death (ED) rate remained relatively high. According to the results of the Surveillance, Epidemiology, and End Results (SEER) database from the USA, treatment advances did not improve the 30-day mortality significantly, being approximately 20 % from 1977 to 2007 [36]. Other results of registries and of clinical trials showed that increasing age, worse performance status, and high WBC count have a negative impact on the ED rate [10, 23, 27].

In clinical trials, the ED rate of elderly APL patients was high and ranged between 10 and 18 % (Table 4) [23, 31–35]. The ED rate observed in population-based registries including unselected patients was even higher. An epidemiologic study from the USA reported an ED rate of 24 % in 502 patients aged over 54 years [28]. In the Swedish Adult Acute Leukemia registry, the ED rate of all 105 registered APL patients (age, 18 to 86 years) was 29 %, and for patients over 60 years, it was 50 % (Table 2) [27]. The German AML Cooperative Group (AMLCG) reported on 91 (30 %) registered patients aged 60 to 83 years among 305 patients with newly diagnosed APL. Of these elderly patients, 25 % were not eligible for inclusion in the studies. The 30-day mortality rate of the non-eligible patients was 48 % [23].

**Table 4:** Outcome in elderly APL patients with ATRA and chemotherapy

Author (year) [reference]	Number of patients	CR (%)	ED (%)	OS (%)	EFS (%)	DFS (%)	CIR (%)	Years
Mandelli (2003) [31]	134	86	12	56	–	59	–	–
Sanz (2004) [32]	104	84	15	–	–	79	9	6
Ades (2005) [33]	129	86	14	58	53	–	16	4
Lataglia (2011) [34]	60	90	10	69	–	65	27	5
Ono (2012 [35])	46	89	11	63	–	65	15	10
Lengfelder (2013) [23]	56	82	18	45	40	48	24	7

*CR* complete remission, *ED* early death, *OS* overall survival, *EFS* event-free survival, *DFS* disease-free survival, *CIR* cumulative incidence of relapse

Detailed analyses of the causes of death indicated that ED in younger APL patients is more frequently due to hemorrhages, whereas in older patients, the causes of ED are more variable including a high rate of death from sepsis or multiorgan failure [22, 23, 27]. The registry reports further demonstrate that age over 60 years was associated with a significantly shorter overall survival mainly influenced by the high rate of ED (Table 2) [27–30].

## Results of clinical studies with ATRA and chemotherapy

Before the introduction of ATRA, 25 to 50 % of patients with APL were probably cured by frontline therapy with anthracyclines and cytosine arabinoside (ara-C) [37]. Investigational studies with chemotherapy before the advent of ATRA showed that increasing cumulative anthracycline doses and the application of maintenance chemotherapy reduced the relapse rate significantly [38, 39]. Older age, microgranular variant, high leukocyte count, and severe coagulopathy were adverse prognostic factors. By combination of anthracycline-based chemotherapy with ATRA, the rate of early death and the frequency of relapses were reduced resulting in a significant improvement of long-term survival and probable cure [37]. It was the aim of subsequent studies to optimize the combination of ATRA and chemotherapy regimens, which were mostly adopted from AML treatment. These studies mainly focused on younger APL patients who had no contraindications against intensive therapy and included only smaller proportions of older patients (Table 1).

Most of the study data of the elderly patients were summarized over longer time periods and were then published in separate reports. The treatment modifications and the main results of larger studies reporting results with ATRA and chemotherapy are listed in Tables 3 and 4. The rate of high-risk patients shows a broad range and is almost lower compared to studies including the whole APL population (Table 1). Furthermore, there is a considerable variability regarding intensity and combination of the chemotherapy regimens. Therefore, a comparison of the study results is problematic. Nevertheless, some conclusions are possible.

In elderly patients treated in these studies, the CR rate was lower (82 to 90 %) and the ED rate higher (11 to 18 %) (Table 4) compared to results of younger study patients mostly reaching CR rates between 90 and 95 % [6, 24]. This may, in part, be explained by the worse tolerance of chemotherapy-related side effects by older patients. Other factors with negative impact on the ED rate are high initial WBC count and very advanced age. In the study of the German AMLCG including 32 % elderly patients with initial WBC count above 10,000/μl, the ED rate in patients with WBC counts below and above 10,000/μl was 8 and 39 %, respectively (*p* = 0.009) [23]. Notably, in patients with high WBC counts aged between 60 and 69 years, the ED rate was 25 %, and in patients 70 years or older, it was 67 %.

It is an important goal in APL treatment to avoid death in CR, particularly due to toxicity of frontline therapy, as relapsed patients have an excellent chance to be cured with salvage regimens [40–42]. The rates of death in CR, which may be in part related to the vulnerability against the chemotherapy, were uniformly higher in elderly patients when compared to younger age groups [23, 31–35]. According to results of the updated French/European APL 93 study, the rate of death in CR was 19 % in patients over 60 years as compared to 6 % in patients aged 18 to 60 years (*p* = 0.05) [43]. The German AMLCG reported a rate of death in CR of 24 % at 10 years in patients over 60 years. In approximately half of these patients, the chemotherapy-related toxicity had influence on the fatal outcome [23]. In an amended Italian ATRA and idarubicin (AIDA) protocol for elderly patients, the second and third consolidation cycle of the original protocol was omitted, and intermittent ATRA maintenance was given to all patients. Compared to the original protocol, the rate of death in CR was reduced from 13 to 8 % [31, 34]. In the Japanese study, in which a relatively dose-intensive regimen was administered, 5 of 36 elderly patients (13 %) died before or during consolidation therapy [35]. The Spanish PETHEMA reported 4 deaths among 86 elderly patients (5 %) during consolidation therapy [32].

Long-term remission and probable cure was achieved in more than 50 % of the elderly patients [23, 31–35]. The rates of overall survival (OS), disease-free survival (DFS), and the cumulative incidence of relapse (CIR) of studies are shown in Table 4. The probability of relapse seems to be influenced by the intensity of the chemotherapy and by the risk profile of the patients. Detailed results of the amended AIDA protocol with reduced chemotherapy showed a moderate increase of the CIR of 27 % compared to 23 % of the historical original protocol, but an improved OS of 76 versus 56 %, respectively [34]. The German AMLCG reported a significantly inferior 7-year OS and CIR of 26 and 58 % in patients with initial WBC counts over 10,000/μl compared to 55 and 13 %, respectively, in patients with lower WBC counts. In 14 selected patients who received treatment intensification with a second induction cycle consisting of age-adapted high-dose ara-C and mitoxantrone (HAM), the DFS was 83 % [23]. Very long-term results of the updated ALP 93 study showed a 10-year OS survival of 77 % of the whole study population compared to 58 % of the elderly group (range of WBC counts, 900 to 1,900/μl) [43].

## ATRA and chemotherapy-based treatment in patients with advanced age over 70 years

There are only few reports on elderly APL patients of advanced age over 70 years. In the report of the APL 93 study, results of 34 (6 %) patients over 70 years were included. Even in these patients, a CR rate of 85 % was achieved [33]. The German AMLCG registered 33 patients over 70 years among 91 patients aged 60 years or older. Of 56 patients treated with 6-thioguanine, ara-C, and daunorubicin (TAD) ± HAM, 18 patients (32 %) were older than 70 years. The CR rate was 89 % in the age group between 60 to 69 years compared to 67 % in patients 70 years or older (*p* = 0.06). The 7-year OS was 54 versus 25 %, respectively (*p* = 0.048), but DFS and CIR showed no difference [23].

From 1999 to 2006, 297 patients aged over 70 years with newly diagnosed AML were seen at a single Canadian center. Among these were 13 patients (4 %) with APL. Their median age was 78 years. Three patients (23 %) had high-risk APL according to the Sanz score. The therapy consisted of ATRA in combination with daunorubicin and ara-C. In patients with impaired left ventricular function, daunorubicin was replaced by amsacrine. The CR rate was 92 %, and the 2-year OS and DFS were 76 and 59 %, respectively [44].

In an Italian multicenter experience, results of 34 patients over 60 years were reported. The median age was 70 years. Twenty-one percent had high risk according to the Sanz score. Twenty-three patients (68 %) received treatment according to the GIMEMA AIDA protocol, and 11 (32 %) had a personalized approach because of bad performance status or cardiomyopathy. In patients treated according to the protocol, the CR rate was 74 %, and in the non-eligible patients, it was 54 %. Ten of 11 patients with ED died from cerebral hemorrhage. The median OS was 38 months [45].

In sporadic elderly patients up to 85 years not qualifying for chemotherapy, ATRA without or combined with low-dose chemotherapy was able to induce remission lasting longer than 3 years. ATRA combined with hydroxyurea for control of leukocytosis is therefore still an option for patients with contraindications against chemotherapy or arsenic trioxide (ATO) [34, 46].

## Treatment based on ATO or gemtuzumab ozogamicin in frontline therapy or relapse

ATO is the most active single agent in treatment of APL. The drug is considerably less toxic compared to approaches with conventional chemotherapy. In particular, treatment-related cytopenia is reduced and cardiotoxicity of anthracyclines can be avoided [40]. The most frequently administered ATO dose was 0.015 μg/kg/day, but lower doses were reported to be effective as well [47]. The combination with ATRA was shown to increase the antileukemic efficacy leading to a faster and more extensive reduction of the leukemic clone compared to ATO alone [48]. In most studies reporting results of ATO in frontline therapy, only small numbers of elderly patients have been included.

Recently, a Chinese group presented the results of 33 elderly patients with newly diagnosed APL aged between 60 and 79 years (median age, 65 years) treated with ATO monotherapy for induction of remission followed by post-remission therapy with intermittent ATO over 4 years. Eighty-five percent had low/intermediate and 15 % had high risk according to the Sanz score. The rate of hematological CR separated into low, intermediate, and high risk was 100, 86, and 80 %, respectively. Three patients died from ED due to hemorrhage. Side effects (CTC grade 3/4), probably related to ATO, were very low. OS, DFS, and cause-specific survival (time from the first day of ATO therapy to death attributed to APL disease or ATO treatment) were 69, 65, and 85 %, respectively [49]. Very recently, an 86-year-old Chinese patient with newly diagnosed APL was reported, who entered molecular remission after 60 days of treatment with low-dose ATO (0.05 mg/kg/day) followed by low-dose ATRA (15 mg/m^2^/day [50]).

In relapsed APL, a number of smaller studies with ATO with or without ATRA were reported. Many studies included elderly patients but did not separately report age-related results [42]. In the US pivotal multicenter study, which included the highest number of patients reported so far, 8 of 40 patients were 60 years or older. Six (75 %) of the older patients and 28 of 32 (88 %) younger patients achieved CR. The 18-month OS of the older patients was 38 % compared to 73 % in patients aged from 18 to 59 years [51]. The different outcome may be influenced by the subsequent stem cell transplantation, which is usually restricted to younger age [52]. According to a historical comparison of French patients with relapsed APL, remission induction with ATO was less toxic compared to conventional therapy with ATRA and chemotherapy, and led to an improved feasibility of transplantation and to a reduction of the transplant-related mortality [40, 53]. Among nine patients from Italy with molecular or hematologic relapse of APL, three were older than 60 years. Eight patients had low/intermediate and one patient had high risk according to the Sanz score. All patients entered molecular remission lasting from 10 months to over 50 months after ATO plus ATRA [54].

Gemtuzumab ozogamicin (GO) is a humanized IgG_4_ anti-CD33 antibody conjugated with calicheamicin, a stable derivative of a natural tumor antibiotic. The unconjugated antibody is relatively untoxic and primarily facilitates the uptake of the calicheamicin derivative into CD33-positive cells. Intracellularly, calicheamicin initiates single- or double-strand DNA breaks, similar to anthracyclines, finally leading to apoptosis and cell death. APL cells have a strong expression of CD33 and are therefore suitable targets for anti-CD33 antibodies including GO [55]. APL cells are further characterized by a low expression of P-glycoprotein (P-170), probably responsible for the particularly high sensitivity to anthracyclines [56, 57].

The clinical efficacy of GO in APL was shown in several reports. In patients with first to fourth molecular relapse after frontline therapy with the AIDA regimen, GO induced remission in 9 (91 %) out of 11 patients tested after two doses of 6 mg/m^2^ and in 13 (100 %) of 13 patients tested after the third dose. The duration of remission was between 3 and over 31 months. GO was administered again in two patients with molecular relapse, and both obtained a new molecular remission [58]. In newly diagnosed APL of younger patients, GO added to the effectiveness of ATRA and could replace anthracyclines in the curative backbone of treatment [59]. Some frail elderly patients were reported, who entered molecular remission after ATRA and GO frontline therapy, even with reduced dose of GO [34, 60, 61].

## Conclusions

Elderly APL patients seem to be as sensitive to therapy as younger patients and should be treated with the intention to reach stabilization or cure, if possible. More than 50 % of patients aged over 60 years included in trials can probably be cured with conventional ATRA and anthracycline-based therapy. Even very elderly or frail patients with contraindication against the common standard therapy have a chance to reach long-term remission or cure with less toxic approaches basing on ATO with or without ATRA or, if applicable, combined with reduced chemotherapy or GO, or even with ATRA alone.

The comparison of registry and study data indicates that the study patients represent a positive selection. Older patients with high initial WBC counts and advanced age have a very high risk and may therefore influence the results of clinical trials negatively due to a higher rate of ED or death in remission. Divergent study results in older age may therefore not only be caused by differences of the treatment regimens but also by the risk profile of the patients. Less intensive chemotherapy has the advantage of a lower rate of treatment-associated mortality and the disadvantage of a higher rate of relapse. Intensive treatment regimens are associated with high antileukemic efficacy but are restricted to selected patients.

Hence, new less toxic approaches with broader applicability are needed to improve the prognosis of the older APL patients. The published data with ATO in elderly patients are limited. A recent Italian–German study randomly compared ATRA and ATO with ATRA and chemotherapy in frontline therapy of low/intermediate-risk patients [62]. The superior outcome of patients assigned to the ATO-plus-ATRA arm and the lower toxicity of this therapy indicate that older patients probably represent a particular target group for ATO-based approaches. This should be investigated in larger clinical trials.
